# An Inquiry into the Difficulties Encountered in the Reduction of Dislocations of the Hip

**Published:** 1897-03

**Authors:** 


**An Inquiry into the Difficulties encountered in the Reduction of Dislocations of the Hip.** By OSCAR H. ALLIS, M.D. Pp. xv., 171. Philadelphia. 1896.

This essay received the award of the Samuel D. Gross prize of one thousand dollars, "awarded every five years to the writer of the best original essay, not exceeding one hundred and fifty printed pages octavo in length, illustrative of some subject in surgical pathology or surgical practice, founded upon original investigations, the candidates for the prize to be American citizens."

The essay is a very valuable one, throwing much new light on the pathology and treatment of dislocations of the hip. The author has done his work in a truly scientific manner, not taking anything for granted, not even the valuable work of his fellow-countryman, Dr. Bigelow, but by experiment on the cadaver and by dissection of pathological specimens he has investigated afresh the various varieties of dislocations and the best way to overcome the difficulties in their reduction. The work is illustrated with 159 diagrams, and although some of them are very crude and somewhat difficult to understand, yet many are very valuable in enabling the reader to easily follow the text. The author would abolish the old nomenclature used in defining the varieties of dislocation of the hip, and would introduce a new classification into inward and outward, each of these being subdivided into low, middle, and high. In reducing the outward form of dislocation he is emphatic in warning us of the danger of making use of the usual method of circumduction; he considers that by this method the sciatic nerve is often hooked up by the head of the femur, the immediate effect of which is a "flexion of the thigh upon the pelvis, and of the leg upon the thigh, owing to the sudden *shortening* of the nerve. The change in the course of the nerve will take up three or four inches of it, and render normal extension impossible." The nerve is thus stretched and flattened over the neck of the femur, its sheath torn, and its fibres separated. He gives examples of this accident happening in his own experience, and we therefore do not doubt the possibility of its occurrence; but the method of reducing outward dislocations of the femur by flexion and circumduction has proved successful in so many hands for so many years, that we should require stronger proof of its danger than we now possess before we willingly discontinued it.

